# Metformin: the updated protective property in kidney disease

**DOI:** 10.18632/aging.103095

**Published:** 2020-05-01

**Authors:** Qingjun Pan, Xing Lu, Chunfei Zhao, Shuzhen Liao, Xiaoqun Chen, Fengbiao Guo, Chen Yang, Hua-feng Liu

**Affiliations:** 1 Key Laboratory of Prevention and Management of Chronic Kidney Disease of Zhanjiang, Institute of Nephrology, Affiliated Hospital of Guangdong Medical University, Zhanjiang 524001, Guangdong, China

**Keywords:** metformin, renal protection, AMPK, lactic acidosis

## Abstract

Metformin is a frontline hypoglycemic agent, which is mainly prescribed to manage type 2 diabetes mellitus with obesity. Emerging evidence suggests that metformin also exerts protective effects against various kidney diseases. Some postulate that kidney disease is actually a metabolic disease, accompanied by nonresolving pathophysiologic pathways controlling oxidative stress, endoplasmic reticulum stress, inflammation, lipotoxicity, fibrosis, and senescence, as well as insufficient host defense mechanisms such as AMP-activated protein kinase (AMPK) signaling and autophagy. Metformin may interfere with these pathways by orchestrating AMPK signaling and AMPK-independent pathways to protect the kidneys from injury. Furthermore, the United States Food and Drug Administration declared metformin is safe for patients with mild or moderate kidney impairment in 2016, assuaging some conservative attitudes about metformin management in patients with renal insufficiency and broadening the scope of research on the renal protective effects of metformin. This review focuses on the molecular mechanisms by which metformin imparts renal protection and its potential in the treatment of various kidney diseases.

## INTRODUCTION

With an aging population, the burden of kidney disease gradually occupies more and more medical resources. Between 2013 and 2016, chronic kidney disease (CKD) was reported in 14.8% of the United States (US) general adult population. As of December 31, 2016, there are 2,160.7 patients with end stage renal disease (ESRD) per 1,000,000 US citizens, according to the US Renal Data System's latest Annual Data Report [1]. Therefore, there is an urgent demand for effective drugs to delay kidney impairment.

Beyond its hypoglycemic action, metformin has pleiotropic protective effects in various disease models, including polycystic ovary syndrome [2], cancer [3], neurological disorders [4], and kidney disease [5]. In particular, emerging evidence has demonstrated potential protective effects of metformin on acute kidney injury (AKI), CKD, diabetic kidney disease (DKD), autosomal dominant (adult) polycystic kidney disease (ADPKD), lupus nephritis (LN), renal neoplasm, and kidney transplantation [6–12].

Metformin protects the kidneys mainly via AMP-activated protein kinase (AMPK) signaling and AMPK-independent pathways. AMPK is a well-known energy and nutrient sensor, which regulates the switch from anabolic to catabolic metabolism to control energy homeostasis [13]. Many kidney diseases are intertwined with abnormal metabolic status, such as hyperglycemia, hyperlipidemia, and hyperuricemia. And many studies has explored the relationship between depressed AMPK activity and kidney disease [14–16], revealing that the AMPK agonist metformin exerts protective actions in a variety of kidney disease models, including those induced by hyperglycemia, advanced glycation end products (AGEs), proteinuria, and high fatty acid and folic acid levels, in both AMPK-dependent and -independent manners. Furthermore, its renal protective efficacy has been partly demonstrated in clinical trials. In this review, we will discuss the renal protective actions of metformin and its therapeutic benefits in different kidney diseases.

## Pharmacological hallmarks of metformin

Metformin is an extract from *Galega officinalis*, which is primarily used to treat type 2 diabetes mellitus (T2DM), with a superior safety and efficacy profile. Metformin exerts a hypoglycemic effect by reducing hepatic glycogenesis and intestinal glucose absorption, improving glucose uptake and utilization in peripheral tissues, and increasing tissue insulin sensitivity [17]. Pharmacokinetic experiments in humans using ^11^C-metformin demonstrated that metformin mainly concentrates in the intestine, liver, and kidneys after oral administration [18]. Metformin is transported into hepatocytes by organic cation transporter 1 (OCT1) and OCT3, and into the renal basolateral membrane by OCT2. Metformin is primarily excreted from the kidneys via multidrug and toxin extrusion 1 and 2 by the means of prototype form [19]. Differences in transporter expression and function lead to pharmacokinetic heterogeneity between individuals, and subsequently, heterogeneous sensitivity to side effects [20]. Although its exact mechanism remains controversial, it is generally accepted that metformin activates AMPK to exert its pharmacological effects via two pathways: one, by promoting the direct phosphorylation of threonine residue 172 of AMPKα by liver kinase B1 (LKB1) [21]; and two, by inhibiting the mitochondrial respiratory chain complex I, resulting in an elevated AMP:ATP ratio and an energy crisis that results in AMPK activation [22]. Upon activation, AMPK coordinates multiple signaling networks to restore the energy balance, with protective effects on renal lesions [23]. However, there are many other mechanisms by which metformin imparts renal protection independently of AMPK, which are discussed in detail below.

## Molecular mechanisms of the renal protective effects of metformin

### Autophagy induction

Autophagy is an evolutionarily conserved catabolic process in eukaryotic cells, in which cells degrade senescent or dysfunctional cytoplasmic components in lysosomes and then reutilize their components. Renal cells maintain a basal level of autophagy under physiological conditions to resist multiple forms of stress. Under pathological conditions, renal cells upregulate autophagy in response to cell stress, but maladaptive autophagy can also induce apoptosis [24]. Current evidence suggests that autophagic flux is generally insufficient in various kidney disease models [25]. Therefore, many researchers are investigating whether modulating autophagy could delay the development of kidney disease.

Li et al. [26] reported that metformin markedly enhanced punctate green fluorescent protein-microtubule associated protein 1 light chain 3 formation in rat renal proximal tubular (NRK-52E) cells, implying that it can influence autophagic flux in the kidneys. Subsequently, Satriano et al. [27] showed that metformin markedly relieved insufficient autophagic flux in the rat kidney cortex in the context of ischemia/reperfusion (IR), and reduced IR damage. In a cisplatin-induced AKI model, pretreatment with metformin decreased apoptosis and induced autophagy in NRK-52E cells. Furthermore, AMPKα small interfering RNA and the autophagy inhibitor 3-methyladenine abrogated the protective effect of metformin on cisplatin-mediated apoptosis, respectively [28]. Additionally, metformin inhibits pronephros cyst formation in polycystin 2-deficient zebrafish by enhancing AMPK-tuberous sclerosis complex-dependent autophagy [29]. These results suggest that metformin-mediated autophagy has beneficial effects.

### Antioxidant properties

Oxidative stress is caused by an imbalance between the production of reactive oxygen species (ROS) and the biological ability to counteract or detoxify their damage through antioxidative mechanisms. Transient increases in ROS protect cells from insult and maintain cellular homeostasis. However, excessive oxidative stress is involved in the pathogenesis of many renal diseases, including DKD [30].

Under sustained hyperglycemic conditions, massive amounts of proteins, lipids, and nucleic acids are glycated through the Maillard reaction to form AGEs. AGEs can induce the expression of oxidative, proinflammatory, and profibrotic mediators in renal cells *via* the advanced glycosylation end-product specific receptor (AGER) [31]. Metformin exerts its antioxidant effect by blocking the AGEs-AGER-ROS axis. Metformin negatively impacts the formation of glyceraldehyde-derived AGEs, protecting proximal tubular epithelial cells from AGEs-mediated injury [32]. In contrast to some scholars’ viewpoints, while metformin treatment reduces AGER expression, it is possible that the positive feedback effect of AGEs on AGER expression is weakened when AGEs generation is inhibited by metformin [33]. Metformin may reduce endogenous ROS generation by inhibiting nicotinamide adenine dinucleotide phosphate oxidase in high glucose-cultured podocytes [34]. In addition, metformin induces the endogenous reductants heme oxygenase 1 (HMOX1) and thioredoxin to reduce ROS generation in high glucose-cultured human kidney proximal tubular (HK-2) cells [35]. Metformin can also block damage cascades downstream of ROS. In an *in vitro* experiment, metformin partly alleviated oxidative stress by inhibiting ROS-induced phosphorylation of p38 mitogen-activated protein kinase (MAPK) in hyperglycemia-stimulated rat glomerular mesangial cells [36].

Aside from DKD, ROS-mediated renal tubular epithelial cell injury is an important risk factor for kidney stone formation. Metformin effectively blunts renal tubular injury resulting from oxalate and renal crystal deposition-mediated lipid peroxidation by attenuating cellular oxidative damage; however, this requires further clinical study [37]. Furthermore, gentamicin-induced nephrotoxicity is partly mediated by mitochondrial oxidative stress, and metformin ameliorated this nephrotoxicity via restoring mitochondrial function and normalizing oxidative stress [38, 39].

Altogether, metformin protects the kidneys in part by blocking ROS generation and signaling pathways downstream of oxidative stress, as well as by increasing antioxidative responses.

### Attenuation of endoplasmic reticulum (ER) stress

ER stress and the course of kidney disease are mutually causal. Albumin overload [40], toxicants [41], and ischemia [42] can result in the accumulation of misfolded and unfolded proteins in the ER, resulting in the activation of ER stress responses to maintain cellular protein homeostasis. Activation of the unfolded protein response (UPR) is a protective response of ER to stress. The UPR inhibits the synthesis of new proteins, improves protein folding ability, and promotes the degradation of misfolded proteins to maintain ER function homeostasis. Notably, chronic or excessive ER stress causes a shift from prosurvival mode to proapoptotic mode, provoking programmed cell death. This occurs through the induction of the proapoptotic ER stress marker C-EBP homologous protein (CHOP), and the activation of the c-jun N-terminal kinase (JNK) and nuclear factor (NF)κB pathways, promoting inflammation, apoptosis, and fibrosis [43, 44]. Therefore, it is worth exploring whether reducing the intensity of ER stress appropriately could alleviate the deterioration of renal function.

Metformin alleviates ER stress-induced renal damage by modulating the UPR [45], partly by inhibiting ROS. Lee et al. revealed that metformin could inhibit ROS-SRC proto-oncogene, non-receptor tyrosine kinase-peroxisome proliferator activated receptor γ-mechanistic target of rapamycin kinase (mTOR) signaling by increasing the expression of endogenous thioredoxin to alleviate albumin-induced ER stress in HK-2 cells. Metformin (1 mM) downregulated glucose-regulated protein 78 (GRP78) and eukaryotic initiation factor 2 α (eIF2α) in HK-2 cells incubated with albumin (5 mg/mL) for 3 days and in the renal tissue of a rat model of proteinuria [46]. Conversely, Allouch et al. showed that cotreatment with metformin (1 mM) and albumin (10 mg/mL) increased GRP78 expression and decreased eIF2α and CHOP expression in NRK-52E cells compared to albumin alone; however, metformin had no effect on GRP78 and CHOP expression in NRK-52E cells treated with 15 mg/mL albumin [47]. The effect of metformin on ER stress may depend on the dose, manner of intervention, and injury severity. Furthermore, it remains unknown how metformin inhibits key molecules (GRP78, eIF2α, and CHOP) in the UPR pathway. Notably, untimely inhibition of the adaptive UPR by metformin can trigger cytotoxic effects [48].

### Anti-inflammatory effects

Metformin may ameliorate renal lesions by abating inflammatory insults. Metformin prevents inflammatory responses through systemic immunomodulation. For example, metformin pretreatment limits immune cell infiltration into renal tissue in unilateral ureteral obstruction (UUO)- and cisplatin-induced models of AKI, thereby reducing inflammatory damage [28, 49, 50]. Christensen et al. [50] reported that metformin regulates the infiltration of microphage subpopulations in renal tissues subjected to three days of UUO. They postulated that metformin reduced microphage infiltration and elevated the ratio of anti-inflammatory M2 macrophages to proinflammatory M1 macrophages to attenuate inflammation damage in the UUO model. However, this notion should be validated using more specific biomarkers to identify microphage subtypes. Additionally, metformin reduces immune cell infiltration into the pronephric ducts of polycystin 2-deficient zebrafish, reducing inflammation-mediated cystogenesis and interfering with PKD progression [29]. In addition to modulating immune cell infiltration into renal tissue, metformin also inhibits their proinflammatory functions. For instance, it reduces the mRNA levels of proinflammatory cytokines (such as interleukin (IL)-1β, IL-6, and tumor necrosis factor α in AGEs-treated bone marrow-derived macrophages via the AMPK-NFκB pathway [51], as well as in the renal tissue of the UUO model [50]. Gu et al. [52] revealed that metformin inhibited NFκB activation and the generation of proinflammatory cytokines (such as monocyte chemoattractant protein 1, intercellular cell adhesion molecule 1, and transforming growth factor β1 (TGFβ1)) in high glucose-treated rat glomerular mesangial cells in vitro. In short, metformin reduces inflammation-induced renal injury by modulating immune cell infiltration and function.

### Attenuation of lipotoxicity

Obese patients are more prone to kidney damage, partly because excess lipids ectopically accumulate in the renal tissue, resulting in lipotoxicity. This contributes to ROS generation, insulin resistance, ER stress, inflammation, and fibrosis [53]. Wang et al. [54] reported that metformin suppressed fatty acid synthesis and deposition to alleviate renal lipotoxicity in Otsuka Long-Evans Tokushima Fatty rats. Combination therapy with metformin and omega-3 polyunsaturated fatty acids improved lipid metabolism in rats with diabetes mellitus (DM) [55]. Besides regulating lipid metabolism, metformin protected mesangial cells from lipotoxicity-induced apoptosis in a diabetic nephropathy model, partially by upregulating glucagon like peptide 1 receptor [56].

### Antifibrotic effects

The kidneys initiate defense responses against various injuries, and maladaptive repair processes promote the phenotypic transformation of renal cells, the proliferation of renal fibroblasts, and abnormal extracellular matrix deposition. As a result, the functional nephron is gradually replaced by connective tissue, driving the formation of interstitial fibrosis, microvascular rarefaction, and even glomerulosclerosis [57]. No effective option exists to reverse renal fibrosis; therefore, delaying renal fibrosis development is an optimal strategy to protect the residual nephron. Metformin has been suggested to affect fibrosis through several mechanisms.

### TGFβ1/SMAD signaling

Dysregulation of TGFβ1 signaling is implicated in renal fibrosis. Angiotensin, glucose, and oxidative stress induce TGFβ1 overexpression in tubular epithelial cells, macrophages, and renal interstitial fibroblasts. TGFβ1/SMAD signaling increases the transcription of α-smooth muscle actin (SMA), fibronectin, collagen I, and vimentin, and decreases E-cadherin expression to promote renal fibrosis [58]. Metformin attenuated TGFβ1 expression in renal tissues from a folic acid-induced rat model of renal fibrosis [59] and a UUO mouse model [60]. Han et al. [61] revealed that metformin interacts with TGFβ1 via its receptor-binding domain, blocking the binding of TGFβ1 to its receptor, and thereby inhibiting the TGFβ1/SMAD pathway. Notably, metformin inhibited UUO-induced SMAD3 phosphorylation in AMPK alpha2 subunit knockout mice, indicating that its anti-renal fibrosis effect is not completely dependent on AMPK signaling [60].

### Epithelial–mesenchymal transition (EMT)

EMT is a prominent process contributing to renal interstitial fibrosis. It is a phenotypic conversion process in which mature tubular epithelial cells transform into myofibroblasts under pathological conditions, and is characterized by the loss of epithelial markers (e.g., E-cadherin, zonula occludens-1) and the acquisition of mesenchymal markers (e.g., α-SMA, fibronectin, and vimentin) [62]. TGFβ1, angiotensin II and hypoxia are vital risks in renal tubular EMT [63]. TGFβ1-induced EMT is mediated either by SMAD2/3 or via non-SMAD (p38 MAPK, JNK, extracellular signal-regulated kinase (ERK), etc.) signaling pathways [64]. Metformin interferes with the TGFβ1-AMPK-tuberin-EMT pathway by activating AMPK and thereby inhibiting extracellular matrix synthesis and other fibrogenic responses [65]. TGFβ1 induces the expression of immediate-early response genes, such as early growth response 1 (EGR1) [66], which is involved in renal tubular EMT [67]. In a recent study, metformin attenuated TGFβ1-induced EMT by inhibiting EGR1, suggesting that this is one of the potential mechanisms behind the renal protective effects of metformin [68]. Wu et al. [69] postulated that metformin inhibits EGR1 by downregulating microRNA (miR)-34a in high glucose-stimulated rat mesangial cells. Therefore, metformin could regulate EGR1 via two pathways: through an AMPK-miR-34a-sirtuin 1 (SIRT1)-EGR1 axis, and via direct AMPK-EGR1 signaling. Furthermore, metformin promotes HMOX1 and thioredoxin expression to decrease ROS levels, thereby alleviating oxidative response-mediated EMT *in vitro* [35].

### Fatty acid metabolism

Extensive research has demonstrated that tubulointerstitial fibrosis is associated with the reduced expression of genes required for fatty acid oxidation (FAO) in renal tubular epithelial cells. Decreased FAO is proposed to cause energy deficiency and renal fibrosis [70]. Acetyl-CoA carboxylase alpha (ACC) is one of the major regulators of FAO, which acts to increase fatty acid synthesis and decrease its oxidation. AMPK phosphorylates ACC to increase FAO, boosting ATP generation. There is evidence that the antifibrotic effect of metformin is partly dependent on its ability to increase FAO by promoting ACC phosphorylation by AMPK [71].

### Hypoxia inducible factor 1

The hypoxia inducible factor (HIF) pathway is an adaptive response to renal insult; however, sustained HIF activation may promote renal fibrosis in CKD [72]. HIF1 inhibition mitigates glomerular hypertrophy, mesangial expansion, matrix accumulation, and albuminuria excretion in type I diabetic OVE26 mice [73]. HIF1 is a heterodimeric transcription factor that regulates oxygen homeostasis, which consists of the constitutively expressed HIF1β subunit and the oxygen-labile HIF1α subunit. Hypoxia prevents the proteasomal degradation of the HIF1α subunit, which then heterodimerizes with HIF1β to regulate the transcription of genes controlling erythropoiesis, angiogenesis, and nucleoside and energy metabolism [72]. Aside from hypoxia, glucose overload [74], angiotensin II [75], and albuminuria [76] also promote renal fibrosis by stabilizing HIF1α. HIF1 modulates extracellular matrix turnover, activates fibrogenic factors such as tissue inhibitor of metalloproteinases and plasminogen activator inhibitor, and promotes EMT [77]. Moreover, HIF1 can act synergistically with TGFβ1 [78].

Metformin suppresses tubular HIF1α stabilization and protects kidneys from renal injury in Zucker diabetic fatty rats independently of AMPKα signaling. It attenuates mitochondrial respiration and thereby reduces cellular oxygen consumption, subsequently enhancing the proteasomal degradation of HIF1α [79]. Notably, HIF1 promotes renal fibrosis in a cell type- and disease phase-specific manner [80].

**Figure 1:**
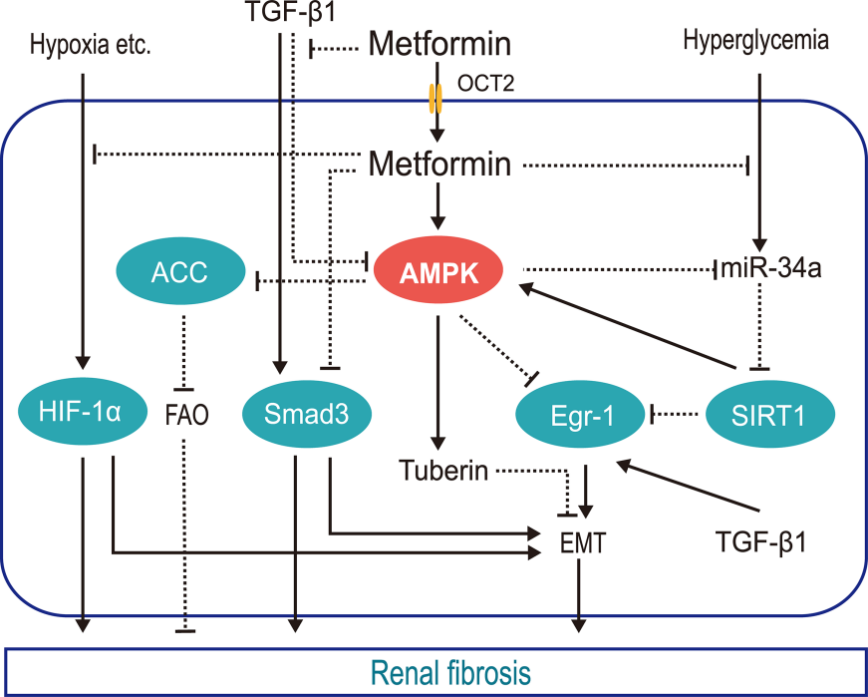
**Schematic of the mechanisms by which metformin protects against renal fibrosis.** Metformin inhibits hypoxia-mediated renal fibrosis by inhibiting HIF1α stabilization via blocking the mitochondrial oxidative respiratory chain, reducing renal oxygen consumption; inhibits TGFβ1 generation and receptor binding to prevent TGFβ1-mediated renal fibrosis; promotes AMPK phosphorylation of ACC to increase FAO and slow renal fibrosis; inhibits hyperglycemia-induced expression of mi-R34a, which negatively regulates AMPK both directly and by downregulating SIRT1, reducing the pro-EMT factor EGR1. (HIF1α, hypoxia inducible factor 1α; TGFβ1, transforming growth factor β1; AMPK, AMP-activated kinase protein; ACC, acetyl-CoA carboxylase; FAO, fatty acid oxidation; miR-34a, microRNA-34a; EGR1, early growth response 1; EMT, epithelial-mesenchymal transition; OCT2, organic cation transporter 2).

Currently, there is no effective way to reverse renal fibrosis; however, protection of the residual nephron is a worthy goal. The antifibrotic effect of metformin requires further clinical validation. The molecular mechanisms controlling this effect are depicted in Figure 1.

### Antiaging effects

Physiological or stress-induced aging weakens the ability of intrinsic renal cells to resist injury and self-repair, increasing AKI risk and accelerating CKD progression. The US FDA has been conducting the randomized double-blind clinical experiment, ‘‘Targeting Aging with Metformin’’ since 2015, extending the uses of metformin into the field of antiaging. Metformin restored expression of the high glucose-downregulates antiaging gene klotho in serum, urine, and renal tissues [81]. Furthermore, metformin downregulates senescence-associated-β-galactosidase and cyclin dependent kinase inhibitors 1A and 2A during hyperglycemia-induced premature aging of glomerular mesangial cells and proximal tubular epithelial cells via the AMPK/mTOR pathway [82, 83]. However, the antiaging effects of metformin have only been demonstrated in some small trails [84], and its precise antiaging effects on the kidneys should be investigated in further basic and clinical studies.

## The pros and cons of metformin: adverse effects and renal protective actions

### Lactic acidosis

**Figure 2:**
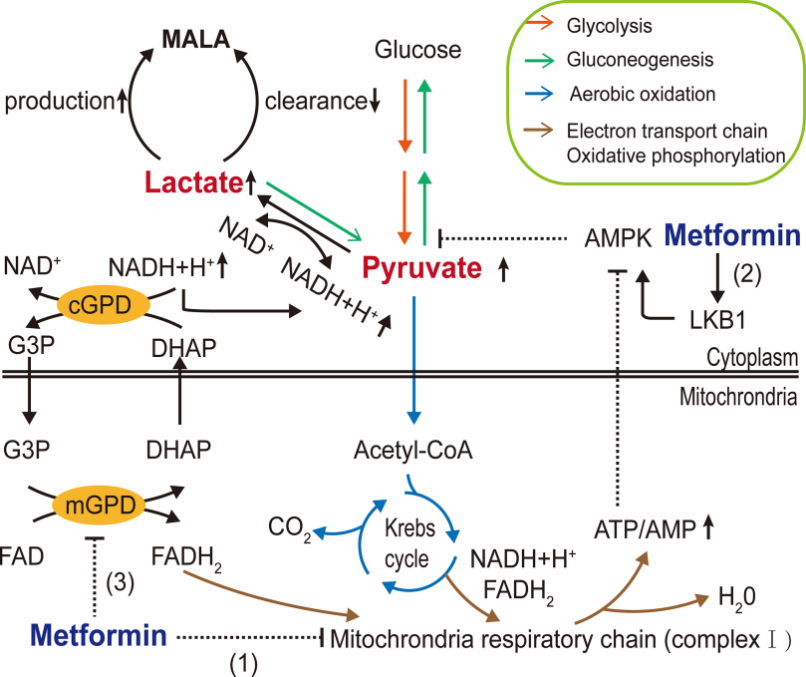
**Molecular mechanisms of metformin-associated lactic acidosis (MALA).** Metformin (1) inhibits mitochondrial respiratory chain complex I, reducing Krebs cycle flux and shifting metabolism toward glycolysis, increasing the pyruvate level; (2) partly inhibits gluconeogenesis through the AMPK pathway, further contributing to pyruvate accumulation and increasing the conversion of accumulated pyruvate to lactate; and (3) inhibits mGPD, blocking the G3P pathway and altering the cytoplasmic redox state, inhibiting the conversion of lactate to pyruvate, resulting in MALA. (AMPK, AMP-activated protein kinase; LKB1, liver kinase B1; G3P, glycerol-3-phosphate; DHAP, dihydroxyacetone phosphate; mGPD, mitochondrial glycerophosphate dehydrogenase; cGPD, cytosolic glycerophosphate dehydrogenase).

The major adverse side effect of metformin exposure is gastrointestinal irritation, including diarrhea, nausea, vomiting, flatulence, and cramps [85]. In addition, the US FDA has given metformin a black box warning regarding lactic acidosis. Because of this, metformin use is restricted for patients with severe renal impairment. Biguanide exposure is related to increased plasma lactic acid levels. Metformin may block the Krebs cycle by inhibiting mitochondrial oxidative respiratory chain complex I, thereby facilitating the Pasteur effect. Lactic acid, a byproduct of glycolysis, accumulates in the body as a result of lactate overproduction or decreased removal [5]. Moreover, in addition to its effects on AMPK-dependent inhibition of gluconeogenesis [86], Madiraju et al. [87, 88] reported that metformin might reduce the conversion of lactate and glycerol to glucose and suppress hepatic gluconeogenesis by inhibiting mitochondrial glycerophosphate dehydrogenase (mGPD) at clinically relevant plasma concentrations. Hence, metformin may interfere with the oxidative pathway and gluconeogenesis pathway of pyruvate, the only precursor of lactate. This would increase the reduction of pyruvate to lactate and decrease the conversion of lactate to pyruvate, resulting in increased theoretical buildup of lactate concentrations (Figure 2). Furthermore, DeFronzo et al. [89] postulated that disease itself exaggerates the risk of metformin-associated lactic acidosis (MALA).

Given the risk of lactic acidosis, how do we weigh the pros and cons of metformin use? MALA is a low probability event. Its incidence varies in different backgrounds, but is generally < 10 per 100,000 patient-years [90]. However, the mortality rate approaches 25% in patients with MALA [91]. In 2016, the US FDA revised metformin safe in patients with mild to moderate renal impairment (estimated glomerular filtration rate (eGFR): 30-60 mL·min^-1^·1.73 m^-2^), but metformin use is contraindicated in patients with eGFR values < 30 mL·min^-1^·1.73 m^-2^. There is no convincing evidence that the label change has increased the MALA rate [92]. The FDA suggests that the initiation and withdrawal of metformin treatment should be based on a comprehensive assessment of the eGFR and risk factors such as hepatic insufficiency, alcoholism, heart impairment, and intra-arterial iodinated contrast exposure. Notably, patient eGFR levels are volatile, and should therefore be periodically monitored [93].

### AKI and CKD

AKI is characterized by a rapid and abrupt reduction of renal function, within days or even a few hours [94], while CKD is defined by a gradual decrease in kidney function over 3 months [95]. However, they are interrelated and cannot simply be classified into two separate diseases. Currently, therapies for AKI and CKD are limited to mitigating etiological factors and treating symptoms, and treatment breakthroughs for these conditions are needed.

Metformin may exert protective effects on AKI and CKD. Li et al. [28] reported that metformin protected a cisplatin-induced AKI model through AMPKα-regulated autophagy induction. Furthermore metformin corrected renal metabolic disorders, suppressed renal fibrosis, and improved renal function in an ablation and infarction rat model of subtotal or five-sixths nephrectomy [96]. Neven et al. [7] revealed that metformin treatment slowed the progression of severe CKD and maintained mineral homeostasis, which reduced the risks of vascular calcification and high bone turnover in CKD-Mineral and Bone Disorder.

In 2017, an observational cohort study of patients with AKI in the Tayside region of Scotland (n= 25,148) revealed that metformin did not affect AKI incidence, but was associated with a 28-day increase in survival [6]. A meta-analysis of 17 observational studies revealed that metformin administration was associated with reduced all-cause mortality in patients with CKD (eGFR= 30-60 mL·min^-1^·1.73 m^-2^) [97].

More recently, Lalau et al. [98] investigated the safety and efficacy of metformin in patients with T2DM and CKD and provided metformin management strategies. The dose-finding study, involving 69 patients, suggested daily doses of 1,500, 1,000, and 500 mg for stages 3A, 3B, and 4 CKD, respectively (Table 1). They suggest that the eGFR and plasma lactate concentration be monitored to evaluate to need to withdraw metformin treatment.

**Table 1:** Metformin management in patients with T2DM with stage 3A, 3B, and 4 CKD.

**CKD stage**	**eGFR (mL·min^-1^·1.73 m^-2^)**	**Recommended daily dose**
3A	45-59	0.5 g in the morning + 1 g in the evening
3B	30-44	0.5 g in the morning + 0.5 g in the evening
4	15-29	500 mg in the morning

T2DM: type 2 diabetes mellitus; CKD: chronic kidney disease; eGFR: estimated glomerular filtration rate.

Taken together, these studies suggest that metformin has renal protective effects against AKI and CKD. However, this notion will require validation through further prospective randomized controlled trials.

### DKD

DKD is a chronic microvascular complication of diabetes mellitus (DM), which is the major cause of ESRD in the US [1]. Reversing high glucose-induced inactivation of AMPK alleviated renal hypertrophy, glomerular basement thickening, podocyte loss, and foot process effacement in OVE26 mice [99], suggesting that AMPK agonist metformin can relieve kidney damage in patients with DM. Aside from its hypoglycemic effect, metformin also delays DKD progression by modulating metabolic dysfunctions, such as insulin resistance, autophagy, oxidative stress, ER stress, inflammation, and renal fibrosis. Insulin resistance, caused by defective insulin signaling in target cells, is a key factor in diabetic glomerulopathy [100]. Podocyte-specific insulin receptor knockout mice develop symptoms of DKD [101]. SIRT1 downregulation in DM is positively associated with insulin resistance [102]. Metformin can overcome hyperglycemia-induced insulin resistance in podocytes by promoting the expression and function of SIRT1 and AMPK [103]. Furthermore, metformin attenuates DM-induced renal medullary tissue hypoxia by inhibiting uncoupling protein 2 in insulinopenic type 1 diabetes rats [104]. As mentioned above, metformin also modulates the interplay between oxidative stress, lipotoxicity**,** fibrosis, and aging in DKD to delay renal exacerbation.

Compared with other hypoglycemic agents, metformin has obvious renal protective functions. For example, compared with sulfonylureas, metformin administration is associated with lower risks of kidney function decline and death, independent of changes in systolic blood pressure, body mass index, and glycated hemoglobin levels over time [105]. An open cohort study of patients with T2DM in primary care (n= 469,688) revealed that metformin decreased the risk of severe T2DM complications, including blindness and severe kidney failure, compared to a group not administered metformin [106]. These results suggest that metformin may be a better choice for DM patients with DKD.

### Autosomal dominant polycystic kidney disease

**Figure 3:**
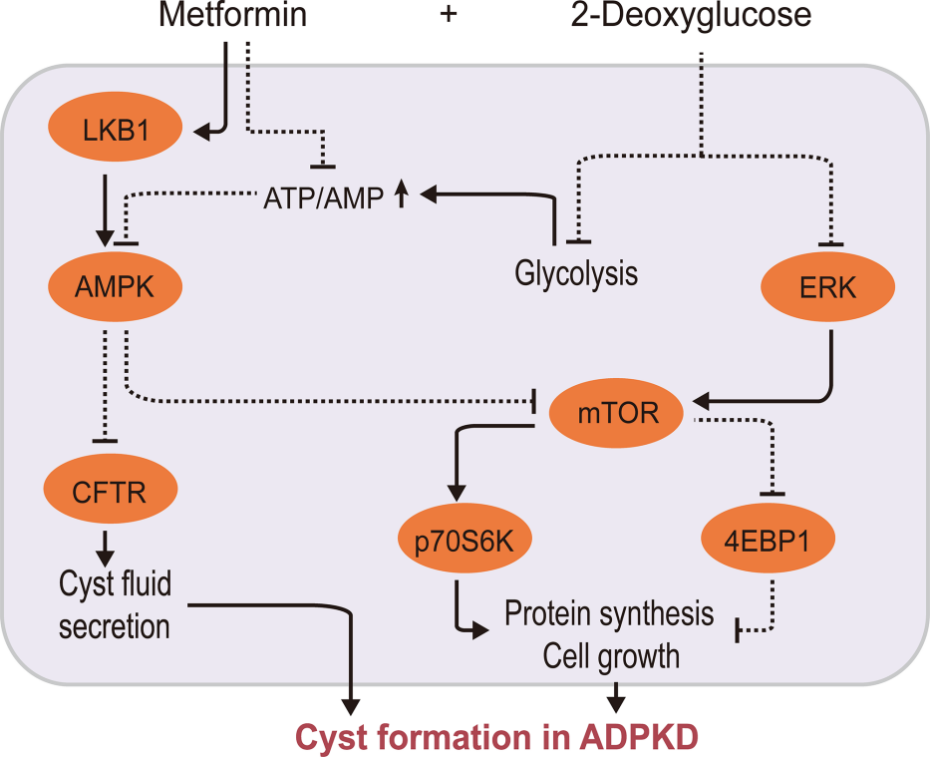
**Metformin/2-deoxyglucose cotreatment delays ADPKD progression.** Metformin interferes with ADPKD cell proliferation by inhibiting CFTR and mTOR signaling via AMPK 2-DG inhibits mTOR via two pathways: by suppressing ERK, an upstream activator of mTOR, and by competitively inhibiting glycolysis, leading to energy imbalance and AMPK activation. This further inhibits CFTR and mTOR, thereby synergistically inhibiting ADPKD proliferation. (2-DG, 2-deoxyglucose; CFTR, cystic fibrosis transmembrane conductance regulator; AMPK, AMP-activated protein kinase; mTOR, mechanistic target of rapamycin kinase; ERK: extracellular signal-regulated kinase; ADPKD, autosomal dominant polycystic kidney disease).

ADPKD is a monogenically inherited cystic kidney disease characterized by renal cysts and extrarenal multisystem manifestations [107]. Cystic cells switch their energy metabolism to glycolysis accompanied with AMPK downregulation, mTOR overactivation, and ERK activation [108, 109]. Therefore, researchers have hypothesized that it may be possible to influence the proliferation and cell cycle progression of cystic cells by regulating their cellular energy metabolism pathways in order to delay ADPKD progression. Cystic fibrosis transmembrane conductance regulator (CFTR) is involved in cyst fluid and electrolyte secretion, and mTOR participates in the proliferation of cyst epithelial cells. Metformin negatively regulates CFTR and mTOR via the AMPK pathway in vitro [110]. Interestingly, metformin and 2-deoxyglucose (2DG), a competitive inhibitor of the rate-limiting glycolytic enzyme hexokinase, synergistically inhibit mTOR signaling to inhibit ADPKD cell proliferation by activating AMPK and suppressing ERK [111, 112], as depicted in Figure 3. Moreover, a few clinical trials have demonstrated the safety and effectiveness of metformin in ADPKD, althought these had small sample sizes [9, 113, 114].

Metformin combined with 2DG may represent a novel intervention strategy for ADPKD, but some researchers have questioned whether clinical trials will have the same inspiring effect, considering the difference in blood drug concentrations between the experimental conditions and those required for clinical treatment [115]. The outcomes of a recent phase 2 placebo-controlled trial regarding the effects of metformin on ADPKD are greatly anticipated.

### LN

Systemic lupus erythematosus (SLE) is a chronic inflammatory autoimmune disease. Aberrant activation of the immune system results in a large number of autoreactive antibodies that attack autoantigens, forming circulating and in situ immune complexes that are deposited in multiple organs, particularly the kidneys.

Metformin exhibits a potential immune regulatory function. Metformin restores immune homeostasis by interfering with T cell subtype differentiation, decreasing autoreactive marginal zone B cells and antibody-secreting plasma cells, and reducing germinal center formation via the AMPK-mTOR-STAT3 pathway in Roquin^san/san^ mice, along with reduced inflammatory mediators and antibodies [116]. A proof-of-concept trial reported that metformin downregulates neutrophil extracellular trap (NETs) mitochondrial DNA (mt-DNA) mediated interferon (IFN)α generation in plasmacytoid dendritic cells (PDCs) to inhibit SLE progression [117]. The dual upregulation of glycolysis and mitochondrial oxidative metabolism is involved in the effector function, activation and proliferation of autoreactive CD4^+^ T cells. Metformin combined with 2DG normalized T cell metabolism, and reversed the lupus phenotype and renal disease in lupus-prone mouse models; however these drugs had no effect alone [118]. Taken together, these studies indicate that metformin improves SLE by inhibiting AMPK/mTOR/STAT3 signaling, NET mtDNA-PDCs-IFNα signaling, and oxidative phosphorylation. However, the efficacy of metformin on lupus nephritis requires further clinical study.

### Renal neoplasms

Renal cell carcinoma (RCC) accounts for 90-95% of renal neoplasms [119]. A recent popular viewpoint has defined cancer as a genetic and metabolic disorder, which establishes high anabolic and catabolic activity to meet proliferation, growth, and survival demands [120]. Consistent with this idea, RCC is accompanied by reduced AMPK levels and dysregulation of proliferation-related mTOR signaling [121]. Therefore, pharmacologically targeting AMPK and mTOR may be a potential therapeutic strategy.

Metformin suppresses RCC progression mainly by promoting apoptosis, as well as inhibiting proliferation and viability in a dose- and time-dependent manner. It has been reported that metformin promotes apoptosis in human RCC (A498) cells [122]. Metformin inhibits RCC cell proliferation and viability by inducing G0/G1 cell cycle arrest [123], and by upregulating cell growth-related miRNAs such as miR-34a [124], miR-26a [125], and miR-21 [126]. Notably, the effects of metformin on apoptosis, G0/G1 phase cell cycle arrest and viability differ between RCC cell lines [127]. Furthermore, metformin promotes RCC cell proliferation under nutrient restriction. Energy stress increases AMPK nuclear translocation, which recruits pyruvate kinase M2 (PKM2) and its downstream effector β-catenin to the nucleus, activating the transcription of proliferation-related genes such as CCND1 and MYC proto-oncogene, bHLH transcription factor [128]. This suggests that combining metformin with a PKM2 inhibitor may be a promising strategy to suppress RCC growth.

In 2017, a meta-analysis (n= 254,329) suggested that metformin administration could improve overall and cancer-specific survival in patients with kidney cancer [129]. A retrospective analysis (n= 1,528 RCC patients) reveled that metformin administration improved survival in patients with localized RCC, but not in those with metastatic RCC [130]. However, a retrospective study (n= 158 patients with diabetes undergoing nephrectomy for kidney cancer) during the same period provided dissenting results [131].

Recently, increasing evidence has revealed a potential therapeutic effect for metformin combined with calorie restriction in colon cancer cell lines or with hemin in breast cancer cell lines [3, 132]. However, the exact action of metformin in renal cancer remains unknown. The antitumor actions of metformin have thus far been demonstrated at experimental doses far exceeding its clinical plasms concentration, sparking speculation as to whether metformin will display clinical effects.

### Renal transplantation

The use of metformin in kidney transplant recipients lacks authoritative clinical criteria. Considering its potential for adverse side effects, metformin requires further assessment regarding the risk of hypoglycemia in these patients. Some studies have indicated that metformin is safe for kidney transplant recipients, improving the survival rate [6, 12, 133–136]. However, due to a series of clinical research limitations, such as small sample sizes and questionable data integrity, the reliability of these studies is unclear. The safety and efficacy of metformin in post-transplantation DM requires further clinical retrospective study and randomized controlled trials with larger sample sizes.

## CONCLUSIONS

Severe kidney damage is irreversible and can develop into ESRD, which requires renal replacement therapy (dialysis or renal transplantation). Kidney transplant surgery is not vigorously promoted because of the rarity of locating matching donors, and early protection of residual nephrons and prevention of further renal deterioration could not only alleviate patient suffering, but also reduce the health and economic burdens of kidney disease worldwide.

In the past few decades, many preclinical and clinical studies have reported the renal protective effects of metformin. At the same time, controversial outcomes of metformin treatment have sparked debate regarding its therapeutic efficacy in some kidney diseases. The renal effects of metformin are complex and dependent on the disease type, as well as the nature and timing of the injury. The clinical efficacy of metformin should be validated in well-designed randomized controlled trials with larger sample sizes, and the precise renal protective mechanisms of metformin should be further explored.
